# Supplementary material for: Flexible and broadband colloidal quantum dots photodiode array for pixel-level X-ray to near-infrared image fusion
Source: Nat Commun. 2023 Sep 2;14:5352. doi: 10.1038/s41467-023-40620-3 (PMC10475073; doi:10.1038/s41467-023-40620-3)
Supplement: Supplementary file 1 — Supplementary Information [file 41467_2023_40620_MOESM1_ESM.pdf]

Supporting information

Flexible and broadband colloidal quantum dots photodiode array for pixel-level X-ray to near-infrared image fusion

Jing Liu[1,2,3], Peilin Liu[1], Tailong Shi[1], Mo Ke[1], Kao Xiong[1], Yuxuan Liu[1], Long Chen[1], Linxiang Zhang[1], Xinyi Liang[1], Hao Li[1], Shuaicheng Lu[1,3], Xinzheng Lan[1,2], Guangda Niu[1,2], Jianbing Zhang[1,2,3], Peng Fei[1,2], Liang Gao[1,2,3]*, Jiang Tang[1,2,4]*

[1] Wuhan National Laboratory for Optoelectronics and School of Optical and Electronic Information, Huazhong University of Science and Technology, 1037 Luoyu Road, 430074, Wuhan, Hubei, China

[2] Optical Valley Laboratory, 430074, Wuhan, P. R. China

[3] Wenzhou Advanced Manufacturing Technology Research Institute of Huazhong University of Science and Technology, 225 Chaoyang New Street, 325105, Wenzhou, P. R. China

[4] National Engineering Research Center for Laser Processing, 1037 Luoyu Road, 430074, Wuhan, P. R. China

* Corresponding authors: highlight@hust.edu.cn, jtang@mail.hust.edu.cn

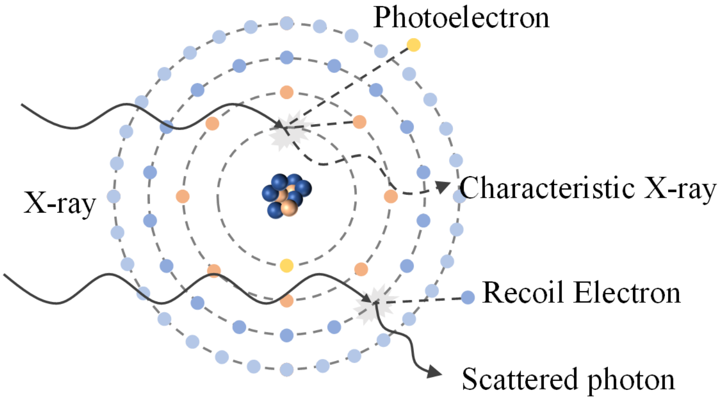

Fig. S1| Mechanism investigation of interactions between X-ray and PbS CQDs film.

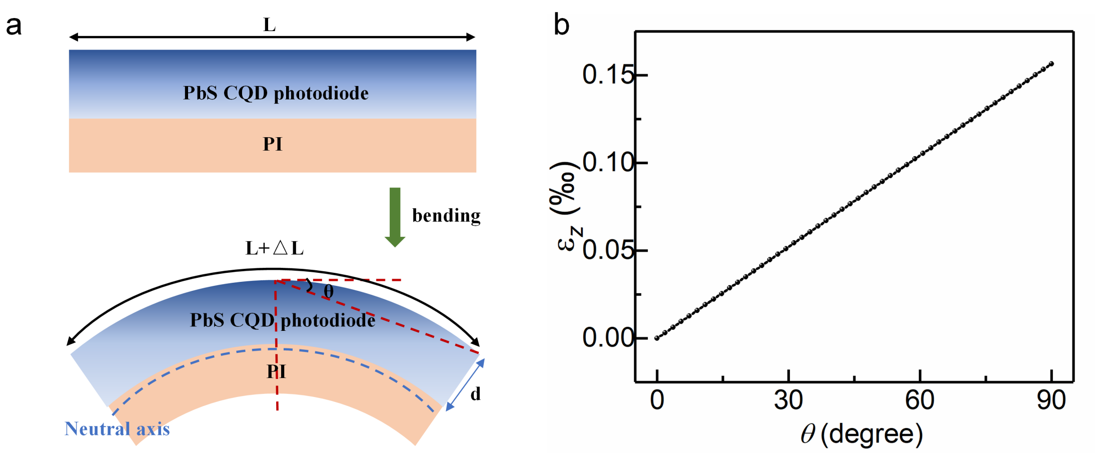

Fig. S2| a, Schematic diagram of bended PbS CQDs photodiode. **b**, Strain as a function of bending curvature.

Analysis of strain within a bended device is shown in Fig. S2a. As the PbS CQDs device is one thousand times thinner than the PI substrate, the neutral plane with zero strain is situated on the surface of the PI substrate[1]. The strain ε_z at different positions can be rewritten as below

$$\varepsilon_z = \frac{z - z_{NA}}{r} \tag{S1}$$

where Z is the location of the CQDs device, Z_{NA} is the location of neutral plane, r is the curvature radius of film. r can be calculated by the equation:

$$r = \frac{360^\circ}{4\theta} \times \frac{l}{2\pi} \tag{S2}$$

where l is the length of the CQDs device, θ is the bending angle. Thus, we can obtain the relationship between θ and ε_z as shown in Fig. S2b. The maximum strain of PbS CQDs device is 0.15‰ at a bending angle of 90°. According to the previous report, the average inter-dot spacing is ~3.21 nm[2]. The inter-dot spacing of PbS CQDs only changes 0.00045 nm. It is possible to achieve a high degree of curvature even when the PbS CQDs are chemically bonded.

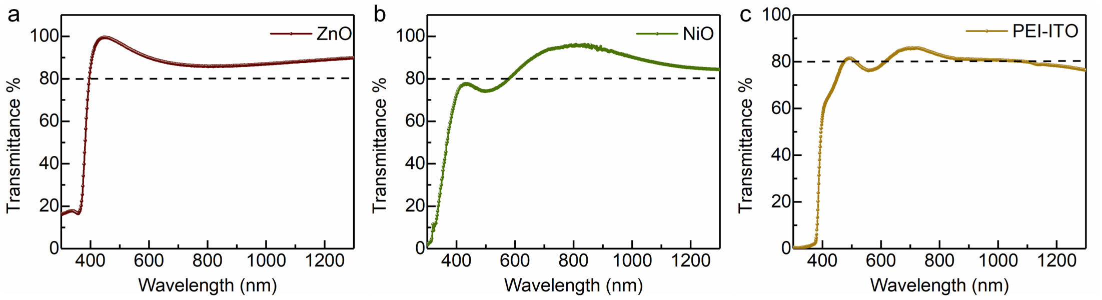

Fig. S3| Transmittance of flexible substrate and transport layers. a, Transmittance of ZnO film with thickness of 120 nm. **b,** Transmittance of NiO_x film with thickness of 40 nm. **c,** Transmittance of polystyrene naphthalate (PEN) with ITO layer.

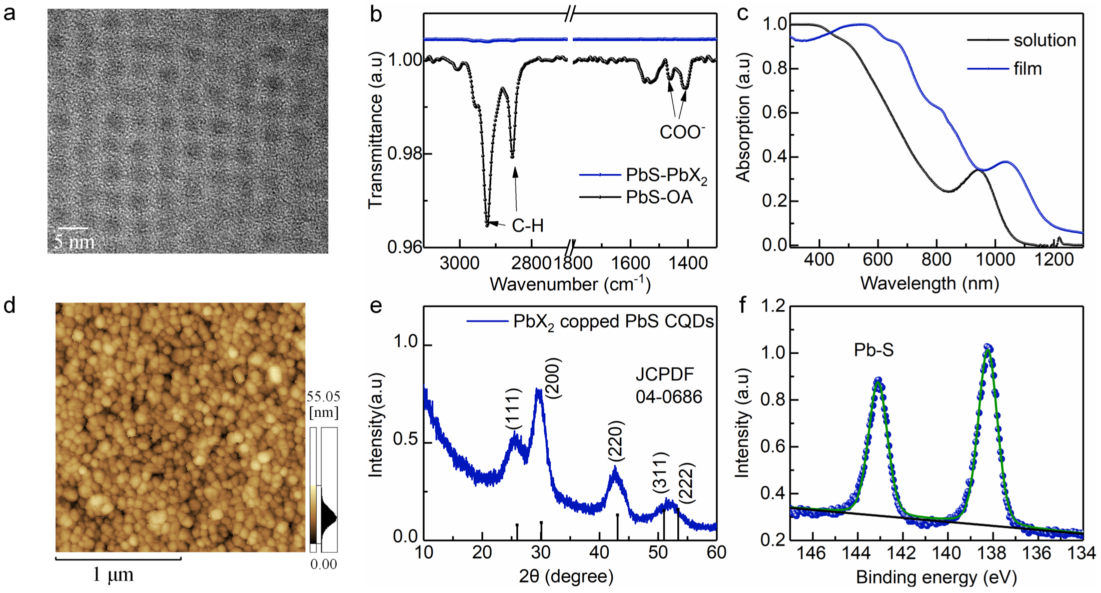

Fig. S4| Characterization of PbS CQDs. a, TEM image of PbS CQDs capped by oleic acid (CQD-OA). **b,** FTIR spectra of CQD-OA and PbX_2 (X=Br, I) capped PbS CQDs (CQD-PbX_2) film. **c,** Absorption spectra of CQD-OA solution and CQD-PbX_2 film. (**d**) AFM image, (**e**) XRD pattern and (**f**) XPS spectrum of CQD-PbX_2 film.

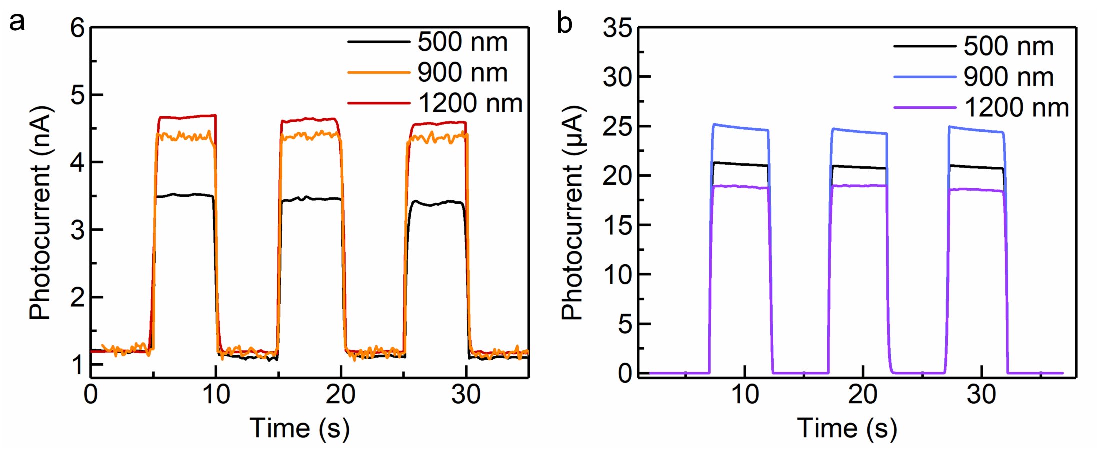

Fig. S5| Photoresponse of PbS CQDs photodiodes with different thickness of PbS CQDs layer. a, Transient responses at –0.1 V bias under 5.1 mGy_{air} s^{-1} dose rates X-ray. **b**, Transient responses at –0.1 V bias illuminated by 970 nm LED with a power density of 0.45 mW cm^{-2}.

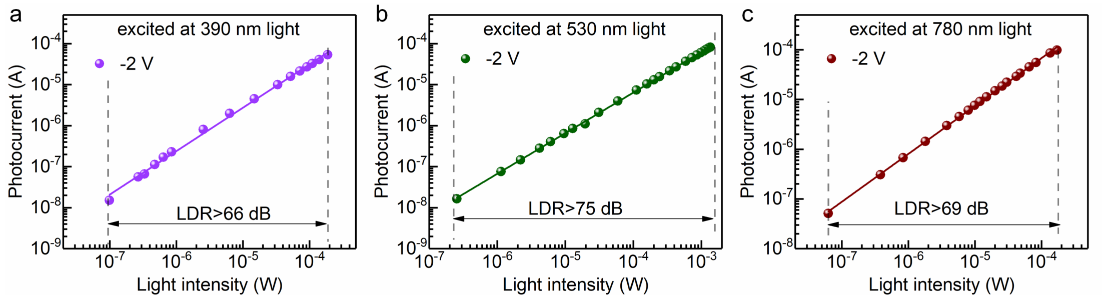

Fig. S6| Linear dynamic range (LDR) of PbS CQDs photodiode at the bias of −2 V under 390 nm (a), 530 nm (b) and 780 nm (c) illumination, respectively. As the photocurrent is linear within the test range, the LDR values are larger than 66 dB, 75 dB and 69 dB, respectively.

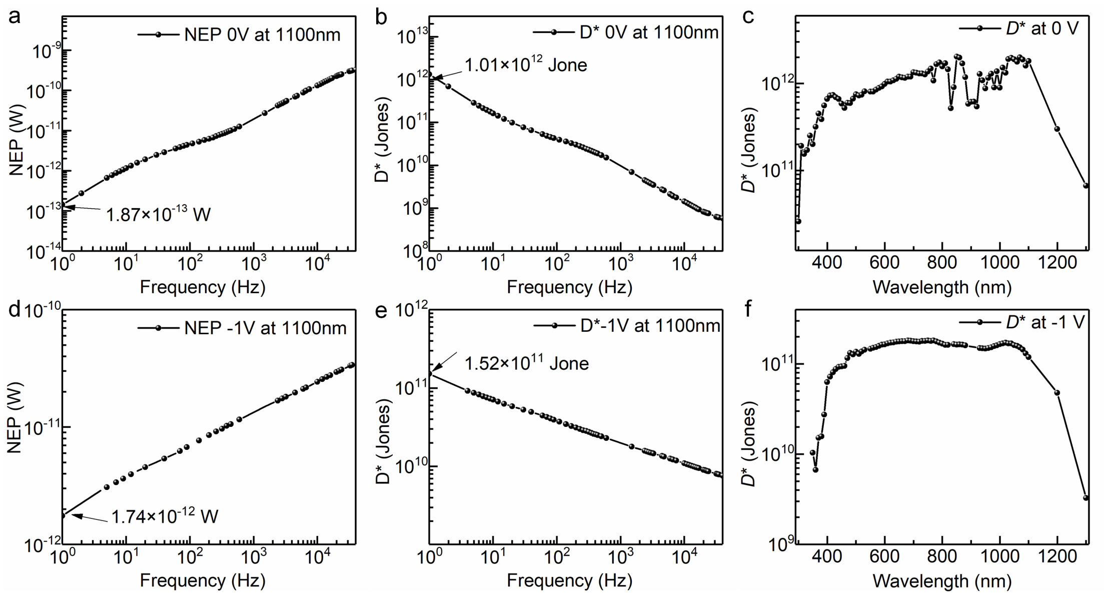

Fig. S7| (a, b) Calculated NEP with normalized bandwidth. NEP is 1.87×10^{-13} W $Hz^{-1/2}$ at zero bias and 1.74×10^{-12} W at −1 V, respectively. (**d, e**) Measured D* as a function of frequency at zero bias and −1 V. D* at 1 Hz is about 1.01×10^{12} Jones at zero bias and 1.52×10^{11} Jones at −1 V, respectively. (**c, f**) Measured D* as a function of wavelength at zero bias and −1 V.

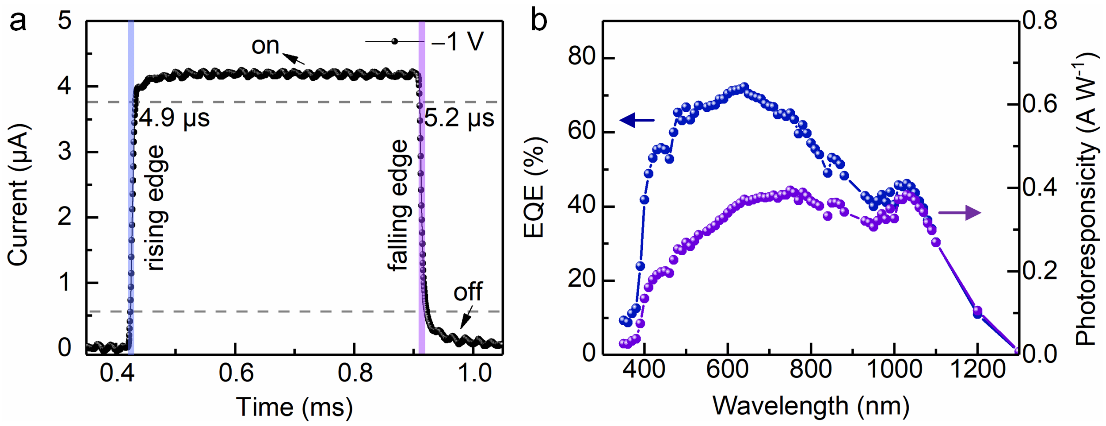

Fig. S8| Device performance of PbS CQDs photodiode under UV-Vis-NIR illumination. a, Temporal response of PbS CQDs photodiode at −1 V under 780 nm illumination. The rising and falling time are 4.9 and 5.2 μs. **b,** EQE and responsivity spectra of PbS CQDs photodiode at −1 V.

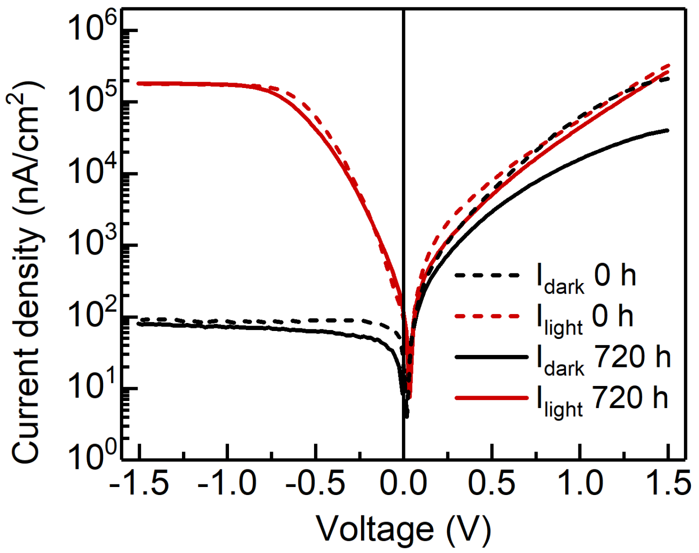

Fig. S9| Stability of PbS CQDs photodiode. The photocurrent and dark current density show negligible change after storing the device in dry air for 720 h.

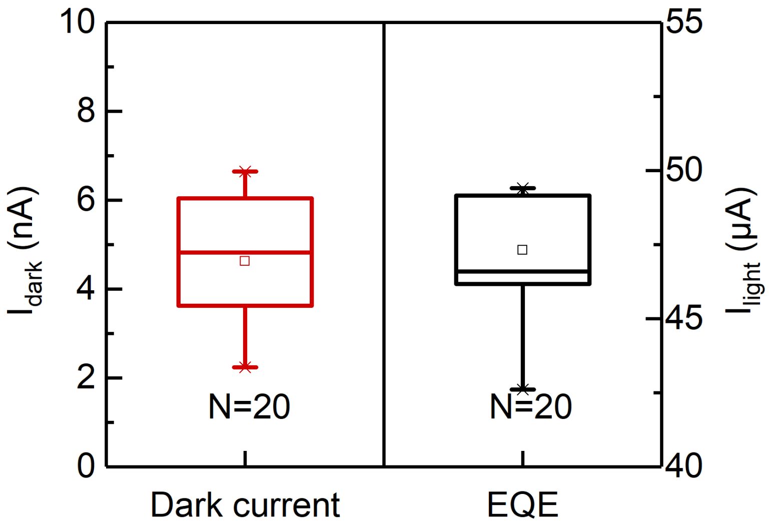

Fig. S10| Homogeneity of PbS CQDs photodiodes. N represents the number of device.

Table S1| Performance list of flexible photodetectors.

material	Spectral range (nm)	R (A/W^{-1})/ EQE (%)	Dark current density (nA/cm^2)	Detectivity (Jones)	Response time (s)	LDR (dB)	Ref.
Sb_2Se_3	450−1050	0.42<br>83%	~900 (–0.1 V)<br>~2000 (–1 V)[C]	2.4×10^{11} [M]	1.6×10^{-6}	95	1
organic	350−1000	1200%	~50 (–1 V)<br>~100 (–5 V) [C]	2.0×10^{12} [M]	/	158	3
$MAPbI_3$	300−800	0.4<br>75%	~34 (-0.1 V)	1.1×10^{10} [M]	9.8×10^{-7}	112	4
ZnO/PbS CQDs	350−1100	4.54	/	3.98×10^{12} [C]	1.01	>60	5
**PbS CQDs**	**350−1300**	**0.38<br>76.6%**	**12.6 (–0.1 V)<br>50.9 (−1 V)**	**1.01×10^{12} [M]**	**5×10^{-6}**	**>85**	**This work**

'C' means the dark current density calculated from the given dark current and device area in the article.

Table S2| The EDS spectra detected from an area of the PbS CQDs film.

Element	Line type	Mass percent (%)	Atomic percent (%)
**Pb**	M	64.78	38.46
**I**	L	20.51	19.88
**S**	K	8.28	31.77
**Br**	L	6.42	9.89

Table S3| The key parameters of flexible X-ray detectors.

Materials	Volume Sensitivity ($\mu C\ mGy^{-1}\ cm^{-3}$)	Bias (V)	Energy (KeV)	Year
PTAA-Bi_2O_3	0.2	200	17.5	2012[6]
P8T2	0.158	50	17	2009[7]
TIPS-pentacene	72	0.2	17	2016[8]
$Cs_2AgBiBr_6$	4	400	45	2018[9]
Bi_2O_3	1712	10	50	2018[10]
Ga_2O_3	271	50	40	2019[11]
$MAPb(I_{0.9}Cl_{0.1})I_3$	362	12	60	2020[12]
$Cs_{0.1}(FA_{0.83}MA_{0.17})_{0.9}Pb(Br_{0.17}I_{0.83})_3$	55.8	0.1	70	2020[13]
SCU-13	13	100	80	2020[14]
Cs_4PbI_6	305	10	30	2021[15]
$FAPbI_3$	284.2	0.5	35	2021[16]
Cs_2TeI_6	217	5	20	2021[17]
Ni-DABDT	19.72	1	26	2021[18]
DABCO-$CsBr_3$	0.533	200	40	2022[19]
Cs_2TeI_6	1512	10	29	2022[20]
$MA_3Bi_2I_6$	206.5	200	30	2022[21]
MPAZE-$NH_4I_3\cdot H_2O$	49.38	20	22	2023[22]
**PbS CQDs**	**200**	**0.1**	**50**	**This work**

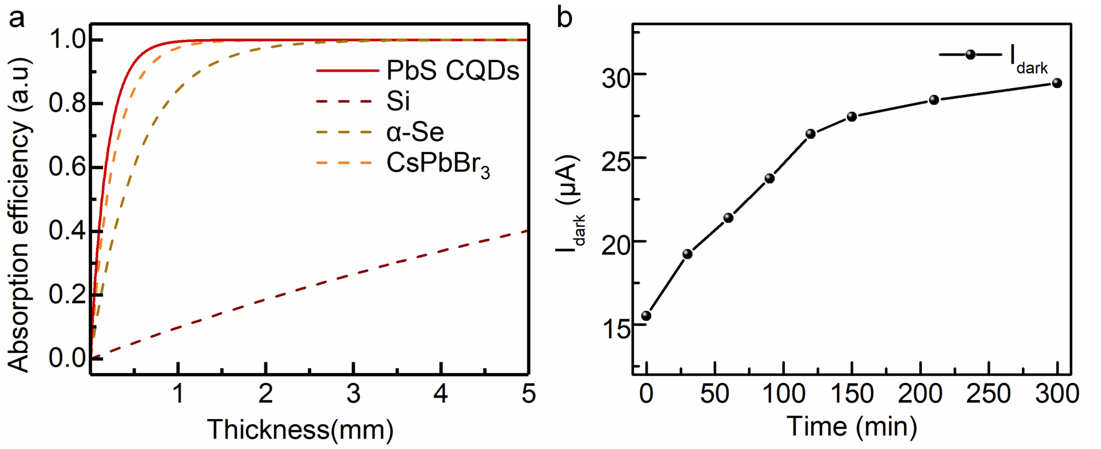

Fig. S11| a, Absorption efficiency of a few semiconductors to 50 keV X-ray photon versus thickness**. b,** Irradiation stability of PbS polycrystalline film prepared by CBD (chemical bath deposition).

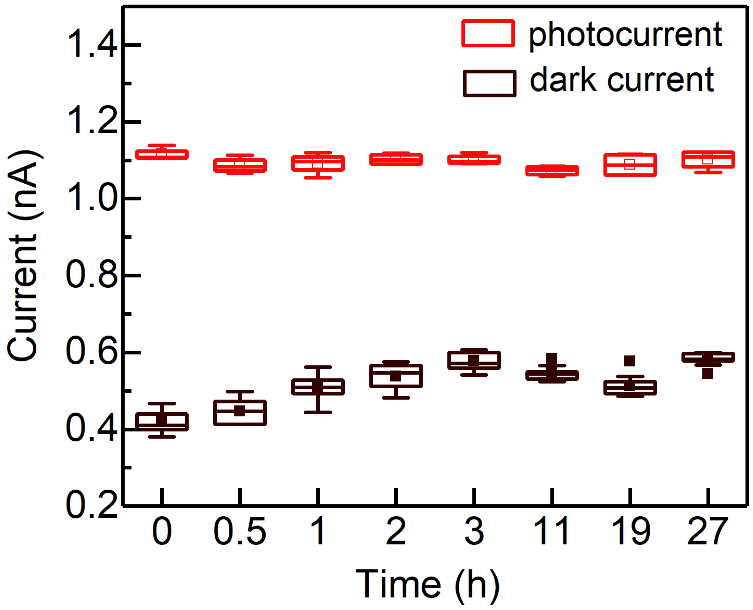

Fig. S12| Stability of PbS CQDs photodiodes under X-ray irradiation.

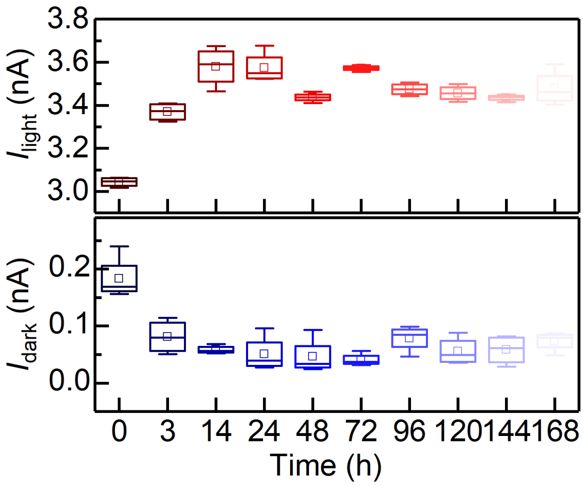

Fig. S13| Stability of PbS CQDs film under X-ray irradiation.

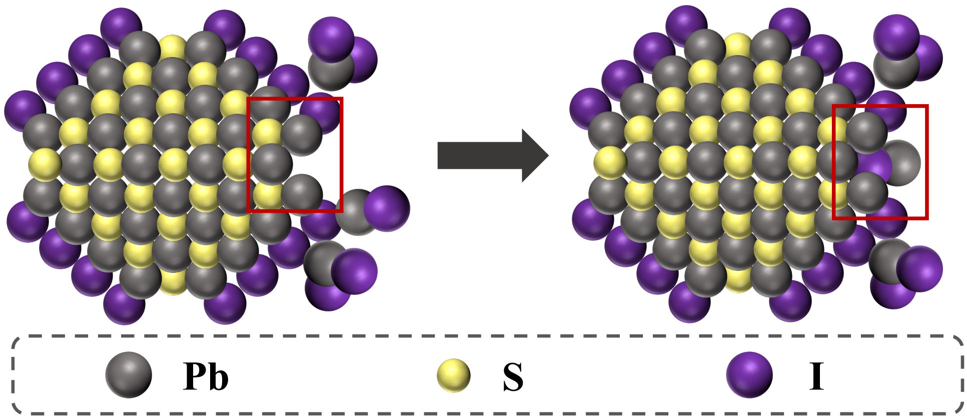

Fig. S14| Schematic diagram of the ligand migration under X-ray irradiation.

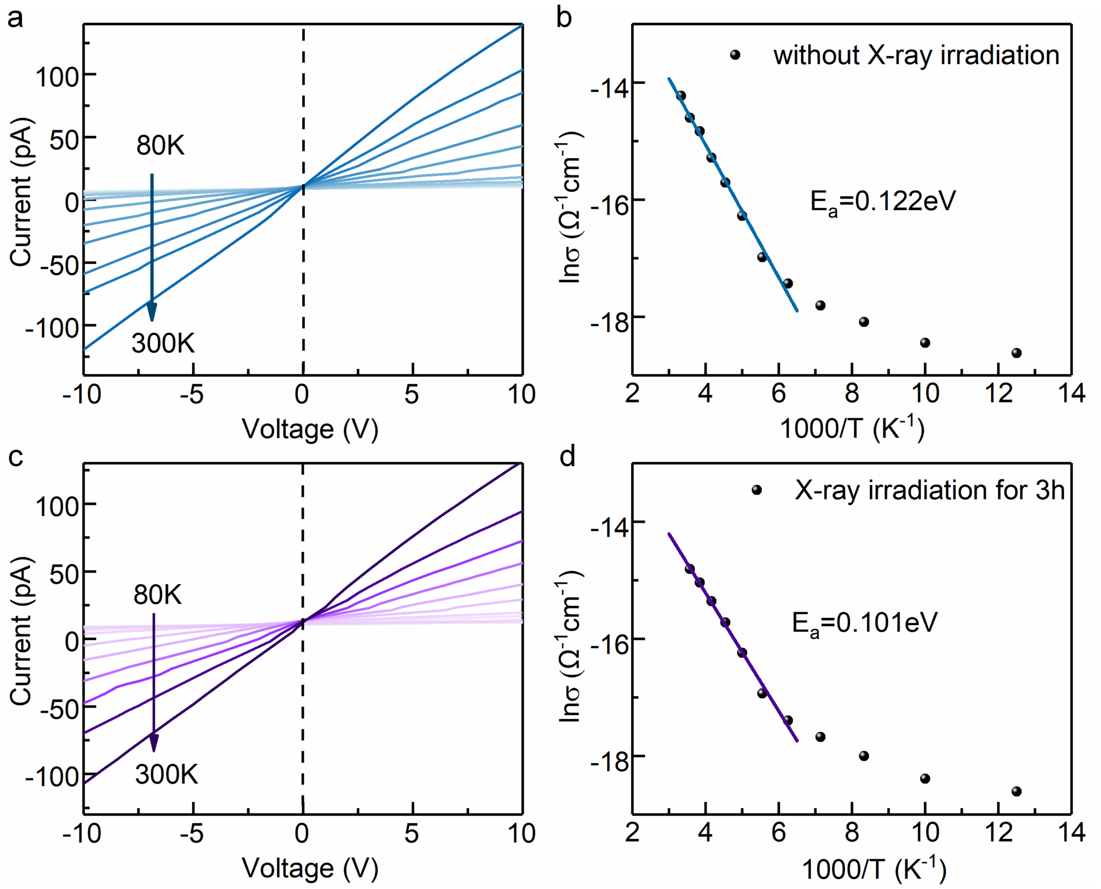

Fig. S15| Defect analysis of PbS CQDs film. (a, c) Current-voltage (*I-V*) curves of Au/PbS CQDs/Au photoconductor without and with X-ray irradiation from 80 K to 300 K, respectively. (**b, d)** Defect depth of PbS CQDs film without and with X-ray irradiation.

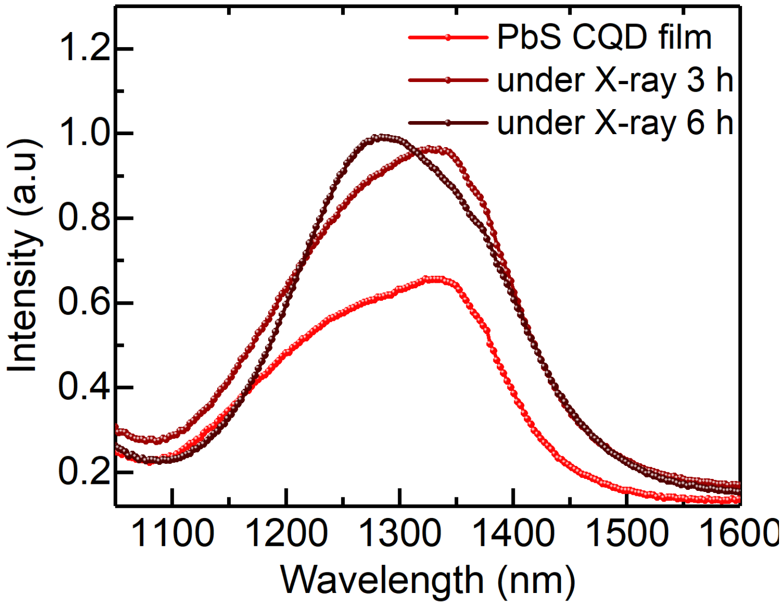

Fig. S16| Photoluminescence of PbS CQDs film under X-ray irradiation.

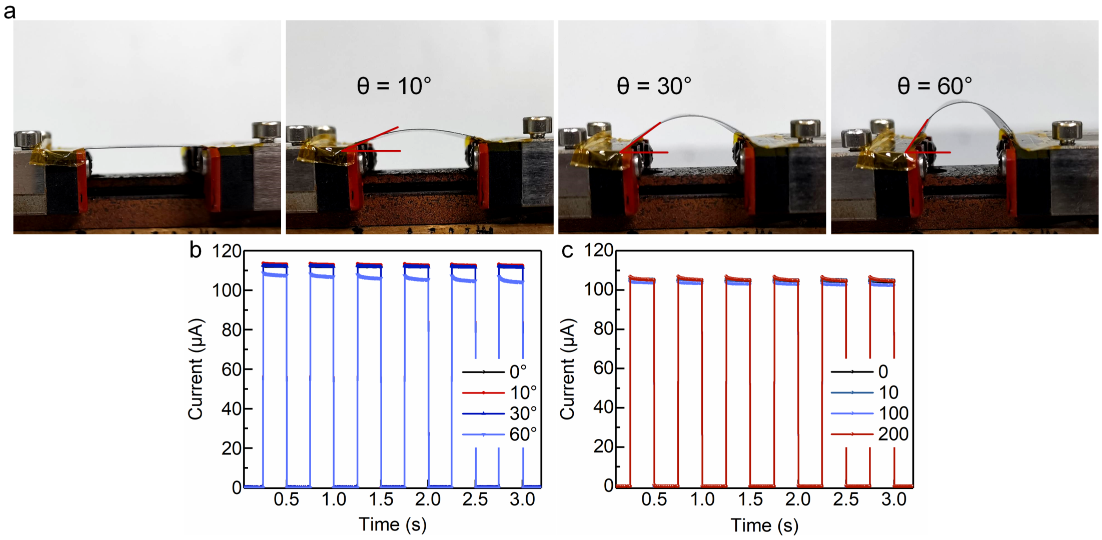

Fig. S17| Flexibility performance of PbS CQDs photodiode. **a,** Photographs of bended PbS CQDs photodiode array at varied angles from 0° to 60°. Photoresponse of flexible PbS CQDs photodiode at different bending angles (**b**) and different bending cycles at a fixed angle of 30° (**c**).

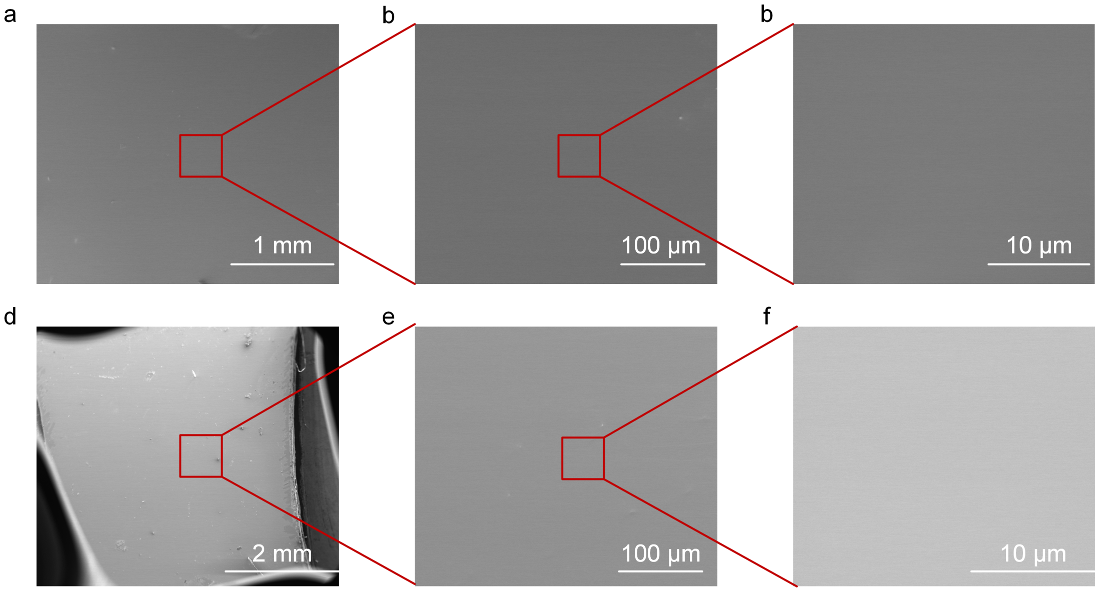

Fig. S18| Flexibility of PbS CQDs film. (**a, b, c**) SEM images of PbS CQDs films before bending with scar bars of 1 mm, 100 µm and 10 µm. (**d, e, f**) SEM images of PbS CQDs films under 30° bending with scar bars of 2 mm, 100 µm and 10 µm.

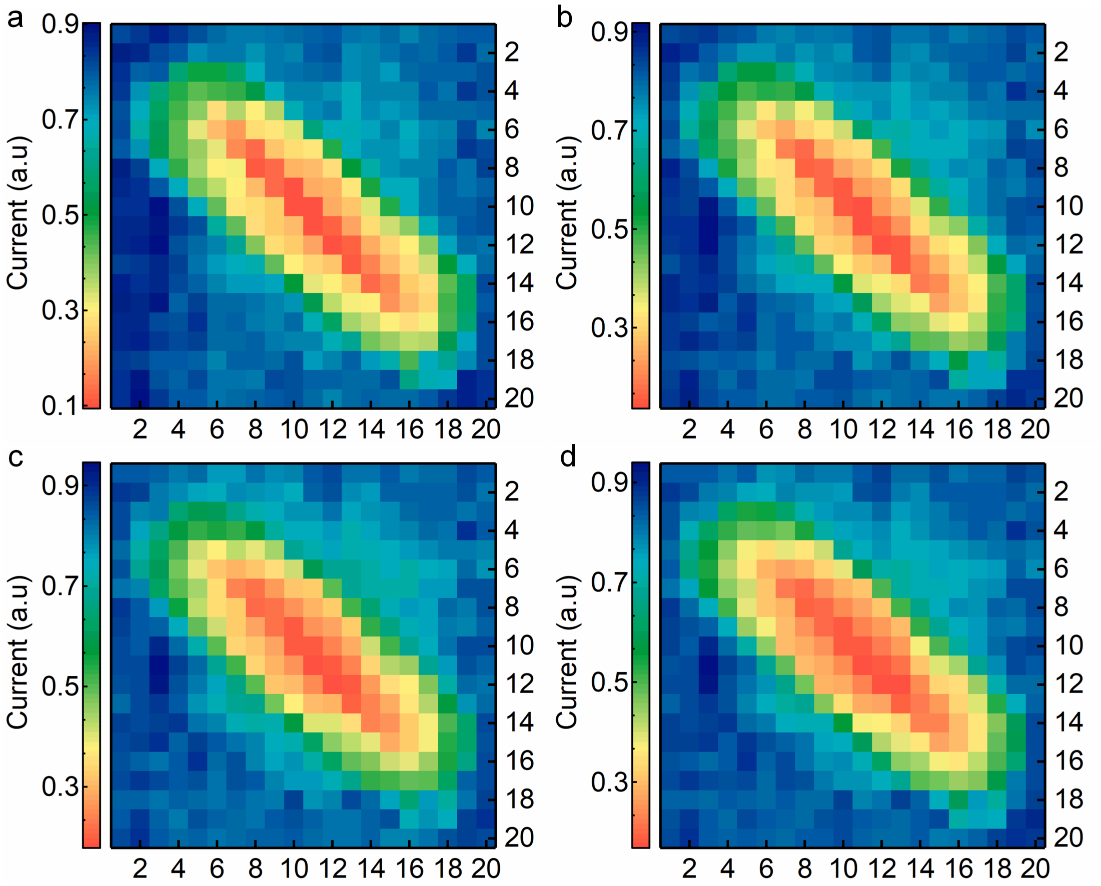

Fig. S19| Fused images with different weights coefficients. a, The weight values of X-ray image, visible image and NIR image are all 0.3. **b,** The weight values of X-ray image, visible image and NIR image are 0.25, 0.25 and 0.5 respectively. **c,** The weight values of X-ray image, visible image and NIR image are 0.16, 0.16 and 0.68 respectively. **d,** The weight values of X-ray image, visible image and NIR image are 0.25, 0.125 and 0.625 respectively.

Supplementary References

1. Chen, C. et al. One-Dimensional Sb2Se3 Enabling a Highly Flexible Photodiode for Light-Source-Free Heart Rate Detection. *ACS Photonics* 7, 352−360 (2020).
2. Liu, M. et al. Hybrid organic-inorganic inks flatten the energy landscape in colloidal quantum dot solids. *Nat. Mater.* 16, 258−263 (2017).
3. Zhao, Z. et al. Photomultiplication Type Broad Response Organic Photodetectors with One Absorber Layer and One Multiplication Layer. *J. Phys. Chem. Lett.* 11, 366-373 (2020).
4. Li, C. et al. Ultrafast and broadband photodetectors based on a perovskite/organic bulk heterojunction for large-dynamic-range imaging. *Light Sci. Appl.* 9, 31 (2020).
5. Peng, M. et al. High-performance flexible and broadband photodetectors based on PbS quantum dots/ZnO nanoparticles heterostructure. *Sci. China Mater.* 62, 225−235 (2018).
6. Intaniwet, A., Mills, C.A., Shkunov, M., Sellin, P.J. & Keddie, J.L. Heavy metallic oxide nanoparticles for enhanced sensitivity in semiconducting polymer x-ray detectors. *Nanotechnology* 23, 235502 (2012).
7. Mills C A, Intaniwet A, Shkunov M, et al. Flexible radiation dosimeters incorporating semiconducting polymer thick films. in Hard X-Ray, Gamma-Ray, and Neutron Detector Physics XI. SPIE. 7449: 300-306 (2009).
8. Basirico, L. et al. Direct X-ray photoconversion in flexible organic thin film devices operated below 1 V. *Nat. Commun.* 7, 13063 (2016).
9. Li, H. et al. Lead-free halide double perovskite-polymer composites for flexible X-ray imaging. *J. Mater. Chem. C* 6, 11961-11967 (2018).
10. Thirimanne, H.M. et al. High sensitivity organic inorganic hybrid X-ray detectors with direct transduction and broadband response. *Nat. Commun.* 9, 2926 (2018).
11. Liang, H. et al. Flexible X-ray Detectors Based on Amorphous Ga2O3 Thin Films. *ACS Photonics* 6, 351-359 (2018).
12. Zhao, J. et al. Perovskite-filled membranes for flexible and large-area direct-conversion X-ray detector arrays. *Nat. Photonics* 14, 612-617 (2020).
13. Mescher, H. et al. Flexible Inkjet-Printed Triple Cation Perovskite X-ray Detectors. *ACS Appl. Mater. Interfaces* 12, 15774-15784 (2020).
14. Liang, C. et al. Thermoplastic Membranes Incorporating Semiconductive Metal-Organic Frameworks: An Advance on Flexible X-ray Detectors. *Angew. Chem. Int. Ed.* 59, 11856-11860 (2020).
15. Li, C. et al. Durable Flexible Polymer-Encapsulated Cs4PbI6 Thin Film for High Sensitivity X-ray Detection. *Nano Lett.* 21, 10279-10283 (2021).
16. Li, W. et al. Evaporated Perovskite Thick Junctions for X-Ray Detection. *ACS Appl. Mater. Interfaces* 13, 2971-2978 (2021).
17. Guo, J. et al. High-Stability Flexible X-ray Detectors Based on Lead-Free Halide Perovskite Cs(2)TeI(6) Films. *ACS Appl. Mater. Interfaces* 13, 23928-23935 (2021).
18. Li, Z. et al. Flexible Lead-Free X-ray Detector from Metal-Organic Frameworks. *Nano Lett.* 21, 6983-6989 (2021).
19. Cui, Q. et al. Efficient Eco-Friendly Flexible X-ray Detectors Based on Molecular Perovskite. *Nano Lett.* 22, 5973-5981 (2022).
20. Guo, J. et al. Oriented preparation of Large-Area uniform Cs2TeI6 perovskite film for high performance X-ray detector. *J. Colloid Interface Sci.* 624, 629-636 (2022).

21. Liu, X. et al. Molecular Doping of Flexible Lead-Free Perovskite-Polymer Thick Membranes for High-Performance X-Ray Detection. *Angew. Chem. Int. Ed.* 61, e202209320 (2022).
22. Li, Z. et al. Hydrogen Bonds Strengthened Metal-Free Perovskite for Degradable X-ray Detector with Enhanced Stability, Flexibility and Sensitivity. *Angew. Chem. Int. Ed.* 62, e202218349 (2023).
